# Supplementary material for: TRPV4 is the temperature-sensitive ion channel of human sperm
Source: eLife. 2018 Jul 2;7:e35853. doi: 10.7554/eLife.35853 (PMC6051745; doi:10.7554/eLife.35853)
Supplement: Figure 1—source data 1. — (A) Inward and Outward monovalent DSper current densities (pA/pF) recorded at -80mV and +80mV, respectively. (B) Divalent CatSper inward current densities (pA/pF). [file elife-35853-fig1-data1.docx]

Source File: Figure 1

**(A) Inward and Outward DSper currents**

| Fig. no. | Experimental condition | at -80 mV, pA/pF | at +80 mV, pA/pF | I_DSper_ -fold change at -80 mV | n, no. of cells | No. of donors |
| --- | --- | --- | --- | --- | --- | --- |
| 1A-C | Baseline (noncap.) | -3.01523 ± 0.33427 | 7.16191 ± 0.4117 |  | 43 | 3 |
| 1A-C | DVF (noncap.) | -17.36211 ± 2.64467 | 126.54189 ± 11.29272 |  | 47 | 3 |
| 1A-C | DVF + 1 mM MG^2+^ (noncap.) | -4.49913 ± 0.41333 | 28.80866 ± 1.93334 |  | 49 | 3 |
| 1A-C | Baseline (cap.) | 10.28051 ± 2.22536 | 15.92858 ± 1.50868 |  | 4 | 3 |
| 1A-C | DVF (cap.) | 49.80851 ± 3.97713 | 183.72758 ±  20.10993 |  | 4 | 3 |
| 1A-C | DVF + 1 mM MG^2+^ (cap.) | 25.57923 ± 5.88305 | 42.66914 ± 9.27394 |  | 4 | 3 |
| 1D-F | DVF + 1 mM MG^2+^ (noncap.) |  |  | 1 | 9 | 3 |
| 1D-F | DVF + 1 mM MG^2+^ + 1 μM NNC (noncap.) |  |  | 0.9688 ± 0.11394 | 9 | 3 |
| 1D-F | DVF + 1 mM MG^2+^ (cap.) |  |  | 1 | 4 | 3 |
| 1D-F | DVF + 1 mM MG^2+^ + 1 μM NNC (cap.) |  |  | 0.95323 ± 0.04992 | 4 | 3 |

**(B) Divalent CatSper inward currents**

| Fig. no. | Extracellular cation | at -80 mV, pA/pF | n, no. of cells | No. of donors |
| --- | --- | --- | --- | --- |
| 1 – Suppl. Fig. 1 | 2 mM Ca^2+^ | 4.53883 ± 1.13556 | 3 | 3 |
| 1 – Suppl. Fig. 1 | 10 mM Ba^2+^ | 24.23848 ± 5.8756 | 3 | 3 |
| 1 – Suppl. Fig. 1 | 50 mM Mg^2+^ | 2.20082 ± 0.26389 | 3 | 3 |
