# Supplementary material for: TRPV4 is the temperature-sensitive ion channel of human sperm
Source: eLife. 2018 Jul 2;7:e35853. doi: 10.7554/eLife.35853 (PMC6051745; doi:10.7554/eLife.35853)
Supplement: Figure 2—source data 1. [file elife-35853-fig2-data1.docx]

Source File: Figure 2

**DSper inward currents as a function of temperature**

| Fig. no | Bath temperature, ˚C | at -80 mV, pA/pF | IDSper normalized to 22 ˚C | n, no. of cells | No. of donors |
| --- | --- | --- | --- | --- | --- |
| 2 A-D (noncap.) | 22 | -3.80175 ± 0.50505 | 1 | 13 | 3 |
| 2 A-D (noncap.) | 24 | -4.31443 ± 0.49468 | 1.189 ± 0.06932 | 13 | 3 |
| 2 A-D (noncap.) | 26 | -4.76471 ± 0.6196 | 1.26194 ± 0.08297 | 11 | 3 |
| 2 A-D (noncap.) | 28 | -5.25178 ± 0.74169 | 1.5585 ± 0.18335 | 12 | 3 |
| 2 A-D (noncap.) | 32 | -5.96155 ± 0.78171 | 1.78391 ± 0.2101 | 11 | 3 |
| 2 A-D (noncap.) | 37 | -8.90887 ± 1.51769 | 2.66874 ± 0.45011 | 11 | 3 |
| 2 A-D (noncap.) | 43 | -8.69134 ± 1.11762 | 3.12259 ± 0.40313 | 6 | 3 |
| 2 A-D (cap.) | 22 | -21.37293 ± 5.63189 | 1 | 3 | 3 |
| 2 A-D (cap.) | 28 | -29.30791 ± 9.32548 | 1.3239 ± 0.09833 | 3 | 3 |
| 2 A-D (cap.) | 32 | -34.60985 ± 10.1326 | 1.57788 ± 0.08802 | 3 | 3 |
| 2 A-D (cap.) | 37 | -45.19159 ± 15.36459 | 2.00596 ± 0.21344 | 3 | 3 |
| 2 A-D (cap.) | 43 | -39.15321 ± 20.56878 | 2.12353 ± 0.36683 | 3 | 3 |
