# Supplementary material for: TRPV4 is the temperature-sensitive ion channel of human sperm
Source: eLife. 2018 Jul 2;7:e35853. doi: 10.7554/eLife.35853 (PMC6051745; doi:10.7554/eLife.35853)
Supplement: Figure 3—source data 1. [file elife-35853-fig3-data1.docx]

Source File: Figure 3

**DSper sodium inward currents**

| Fig. no | Experimental condition | at -80 mV, normalized to control | at -80 mV, normalized to 22 ̊C | n, no. of cells | No. of donors |
| --- | --- | --- | --- | --- | --- |
| 3 B-C | DSper control | 1 |  | 3 | 2 |
| 3 B-C | + 1 μM NNC | 0.91146 ± 0.10078 |  | 3 | 2 |
| 3 D-E | 22 ̊C |  | 1 | 3 | 2 |
| 3 D-E | 27 ̊C |  | 1.4069 ± 0.08929 | 3 | 2 |
| 3 D-E | 32 ̊C |  | 2.21529 ± 0.39038 | 3 | 2 |
| 3 D-E | 37 ̊C |  | 4.07356 ± 0.50788 | 3 | 2 |
| 3 D-E | 42 ̊C |  | 4.44307 ± 0.97919 | 3 | 2 |
