# Supplementary material for: TRPV4 is the temperature-sensitive ion channel of human sperm
Source: eLife. 2018 Jul 2;7:e35853. doi: 10.7554/eLife.35853 (PMC6051745; doi:10.7554/eLife.35853)
Supplement: Figure 4—source data 1. — (A) DSper inward currents potentiated by RN1747. (B) DSper currents after stimulation with Capsaicin. (C) Calcium imaging (stimulation with 10 μM capsaicin after 20 s). (D) Calcium imaging (stimulation with 500 μM carvacrol after 20 s). (E) Pregnonolone sulfate effect on IDSper. [file elife-35853-fig4-data1.docx]

Source File: Figure 4

**(A) DSper inward currents potentiated by RN1747**

| Fig. no | Experimental condition | at -80 mV, normalized to control | at +80 mV, normalized to control | n, no. of cells | No. of donors |
| --- | --- | --- | --- | --- | --- |
| 4 A-B | DSper control | -1 | 1 | 5 | 2 |
| 4 A-B | + 10 μM RN1747 | -1.19601 ± 0.11 | 2.22697 ± 0.23 | 5 | 2 |

**(B) DSper currents after stimulation with Capsaicin**

| Fig. no | Experimental condition | at -80 mV, normalized to control | at +80 mV, normalized to control | n, no. of cells | No. of donors |
| --- | --- | --- | --- | --- | --- |
| 4 – Suppl. Fig. 1 | DSper control | -1 | 1 | 4 | 3 |
| 4 – Suppl. Fig. 1 | + 1 μM Capsaicin | -1.06242 ± 0.06869 | 0.98881 ± 0.05053 | 4 | 3 |
| 4 – Suppl. Fig. 1 | + 10 μM Capsaicin | -1.06637 ± 0.09003 | 0.90554 ± 0.05565 | 4 | 3 |
| 4 – Suppl. Fig. 1 | + 10 μM Capsaicin w/ 30 μM PIP_2_ inside | -0.91182 ± 0.03 | 0.88286 ± 0.07 | 6 | 3 |

**(C) Calcium imaging (stimulation with 10 μM capsaicin after 20 seconds)**

| Fig. no | Bath solution | Time (s) | Mean ΔF/F_0_ | SEM of mean ΔF/F_0_ | n, no. of cells | No. of donors |
| --- | --- | --- | --- | --- | --- | --- |
| 4 – Suppl. Fig. 1 | HS | 1 | 0.03975 | 0.06103 | 5 | 2 |
| 4 – Suppl. Fig. 1 | HS | 2 | 0.00721 | 0.05456 | 5 | 2 |
| 4 – Suppl. Fig. 1 | HS | 3 | 0.06019 | 0.03978 | 5 | 2 |
| 4 – Suppl. Fig. 1 | HS | 4 | 0.10654 | 0.03607 | 5 | 2 |
| 4 – Suppl. Fig. 1 | HS | 5 | 0.07368 | 0.03479 | 5 | 2 |
| 4 – Suppl. Fig. 1 | HS | 6 | 0.04196 | 0.01911 | 5 | 2 |
| 4 – Suppl. Fig. 1 | HS | 7 | 0.02 | 0.02271 | 5 | 2 |
| 4 – Suppl. Fig. 1 | HS | 8 | 0.0106 | 0.01356 | 5 | 2 |
| 4 – Suppl. Fig. 1 | HS | 9 | 0.01194 | 0.01808 | 5 | 2 |
| 4 – Suppl. Fig. 1 | HS | 10 | -0.03452 | 0.00932 | 5 | 2 |
| 4 – Suppl. Fig. 1 | HS | 11 | 0.03559 | 0.01661 | 5 | 2 |
| 4 – Suppl. Fig. 1 | HS | 12 | -4.10685E-4 | 0.02622 | 5 | 2 |
| 4 – Suppl. Fig. 1 | HS | 13 | 0.00149 | 0.01572 | 5 | 2 |
| 4 – Suppl. Fig. 1 | HS | 14 | -0.03309 | 0.03298 | 5 | 2 |
| 4 – Suppl. Fig. 1 | HS | 15 | -0.05512 | 0.01627 | 5 | 2 |
| 4 – Suppl. Fig. 1 | HS | 16 | -0.0177 | 0.02191 | 5 | 2 |
| 4 – Suppl. Fig. 1 | HS | 17 | -0.05919 | 0.02893 | 5 | 2 |
| 4 – Suppl. Fig. 1 | HS | 18 | -0.06283 | 0.04059 | 5 | 2 |
| 4 – Suppl. Fig. 1 | HS | 19 | -0.06378 | 0.04294 | 5 | 2 |
| 4 – Suppl. Fig. 1 | HS | 20 | -0.08231 | 0.02956 | 5 | 2 |
| 4 – Suppl. Fig. 1 | HS + 10 μM capsaicin | 21 | -0.04777 | 0.03526 | 5 | 2 |
| 4 – Suppl. Fig. 1 | HS + 10 μM capsaicin | 22 | -0.01727 | 0.04069 | 5 | 2 |
| 4 – Suppl. Fig. 1 | HS + 10 μM capsaicin | 23 | -0.06404 | 0.03937 | 5 | 2 |
| 4 – Suppl. Fig. 1 | HS + 10 μM capsaicin | 24 | -0.06452 | 0.03658 | 5 | 2 |
| 4 – Suppl. Fig. 1 | HS + 10 μM capsaicin | 25 | -0.0975 | 0.04942 | 5 | 2 |
| 4 – Suppl. Fig. 1 | HS + 10 μM capsaicin | 26 | -0.07044 | 0.05779 | 5 | 2 |
| 4 – Suppl. Fig. 1 | HS + 10 μM capsaicin | 27 | -0.04543 | 0.05984 | 5 | 2 |
| 4 – Suppl. Fig. 1 | HS + 10 μM capsaicin | 28 | -0.05921 | 0.06062 | 5 | 2 |
| 4 – Suppl. Fig. 1 | HS + 10 μM capsaicin | 29 | -0.0911 | 0.05967 | 5 | 2 |
| 4 – Suppl. Fig. 1 | HS + 10 μM capsaicin | 30 | -0.11258 | 0.06772 | 5 | 2 |
| 4 – Suppl. Fig. 1 | HS + 10 μM capsaicin | 31 | -0.08967 | 0.06065 | 5 | 2 |
| 4 – Suppl. Fig. 1 | HS + 10 μM capsaicin | 32 | -0.10343 | 0.06638 | 5 | 2 |
| 4 – Suppl. Fig. 1 | HS + 10 μM capsaicin | 33 | -0.09207 | 0.06196 | 5 | 2 |
| 4 – Suppl. Fig. 1 | HS + 10 μM capsaicin | 34 | -0.09737 | 0.0605 | 5 | 2 |
| 4 – Suppl. Fig. 1 | HS + 10 μM capsaicin | 35 | -0.12326 | 0.0725 | 5 | 2 |
| 4 – Suppl. Fig. 1 | HS + 10 μM capsaicin | 36 | -0.12512 | 0.06763 | 5 | 2 |
| 4 – Suppl. Fig. 1 | HS + 10 μM capsaicin | 37 | -0.07485 | 0.06015 | 5 | 2 |
| 4 – Suppl. Fig. 1 | HS + 10 μM capsaicin | 38 | -0.11177 | 0.06434 | 5 | 2 |
| 4 – Suppl. Fig. 1 | HS + 10 μM capsaicin | 39 | -0.13018 | 0.07074 | 5 | 2 |
| 4 – Suppl. Fig. 1 | HS + 10 μM capsaicin | 40 | -0.14309 | 0.0653 | 5 | 2 |
| 4 – Suppl. Fig. 1 | HS + 10 μM capsaicin | 41 | -0.10673 | 0.07125 | 5 | 2 |
| 4 – Suppl. Fig. 1 | HS + 10 μM capsaicin | 42 | -0.08725 | 0.07686 | 5 | 2 |
| 4 – Suppl. Fig. 1 | HS + 10 μM capsaicin | 43 | -0.11358 | 0.06614 | 5 | 2 |
| 4 – Suppl. Fig. 1 | HS + 10 μM capsaicin | 44 | -0.08626 | 0.0668 | 5 | 2 |
| 4 – Suppl. Fig. 1 | HS + 10 μM capsaicin | 45 | -0.09887 | 0.06884 | 5 | 2 |
| 4 – Suppl. Fig. 1 | HS + 10 μM capsaicin | 46 | -0.07932 | 0.0719 | 5 | 2 |
| 4 – Suppl. Fig. 1 | HS + 10 μM capsaicin | 47 | -0.07951 | 0.08384 | 5 | 2 |
| 4 – Suppl. Fig. 1 | HS + 10 μM capsaicin | 48 | -0.10278 | 0.08167 | 5 | 2 |
| 4 – Suppl. Fig. 1 | HS + 10 μM capsaicin | 49 | -0.09406 | 0.09352 | 5 | 2 |
| 4 – Suppl. Fig. 1 | HS + 10 μM capsaicin | 50 | -0.10076 | 0.0805 | 5 | 2 |
| 4 – Suppl. Fig. 1 | HS + 10 μM capsaicin | 51 | -0.00173 | 0.05178 | 5 | 2 |
| 4 – Suppl. Fig. 1 | HS + 10 μM capsaicin | 52 | -0.10482 | 0.08577 | 5 | 2 |
| 4 – Suppl. Fig. 1 | HS + 10 μM capsaicin | 53 | -0.09059 | 0.09115 | 5 | 2 |
| 4 – Suppl. Fig. 1 | HS + 10 μM capsaicin | 54 | -0.12441 | 0.09078 | 5 | 2 |
| 4 – Suppl. Fig. 1 | HS + 10 μM capsaicin | 55 | -0.07905 | 0.08585 | 5 | 2 |
| 4 – Suppl. Fig. 1 | HS + 10 μM capsaicin | 56 | -0.10103 | 0.09975 | 5 | 2 |
| 4 – Suppl. Fig. 1 | HS + 10 μM capsaicin | 57 | -0.10502 | 0.08535 | 5 | 2 |
| 4 – Suppl. Fig. 1 | HS + 10 μM capsaicin | 58 | -0.11374 | 0.08635 | 5 | 2 |
| 4 – Suppl. Fig. 1 | HS + 10 μM capsaicin | 59 | -0.12941 | 0.08661 | 5 | 2 |
| 4 – Suppl. Fig. 1 | HS + 10 μM capsaicin | 60 | -0.13307 | 0.09629 | 5 | 2 |
| 4 – Suppl. Fig. 1 | HS + 10 μM capsaicin | 61 | -0.09809 | 0.08276 | 5 | 2 |
| 4 – Suppl. Fig. 1 | HS + 10 μM capsaicin | 62 | -0.11242 | 0.08167 | 5 | 2 |
| 4 – Suppl. Fig. 1 | HS + 10 μM capsaicin | 63 | -0.12605 | 0.08772 | 5 | 2 |
| 4 – Suppl. Fig. 1 | HS + 10 μM capsaicin | 64 | -0.11937 | 0.08056 | 5 | 2 |
| 4 – Suppl. Fig. 1 | HS + 10 μM capsaicin | 65 | -0.09768 | 0.08414 | 5 | 2 |
| 4 – Suppl. Fig. 1 | HS + 10 μM capsaicin | 66 | -0.09496 | 0.08138 | 5 | 2 |
| 4 – Suppl. Fig. 1 | HS + 10 μM capsaicin | 67 | -0.09379 | 0.08126 | 5 | 2 |
| 4 – Suppl. Fig. 1 | HS + 10 μM capsaicin | 68 | -0.1165 | 0.08482 | 5 | 2 |
| 4 – Suppl. Fig. 1 | HS + 10 μM capsaicin | 69 | -0.10247 | 0.10279 | 5 | 2 |
| 4 – Suppl. Fig. 1 | HS + 10 μM capsaicin | 70 | -0.0885 | 0.11422 | 5 | 2 |
| 4 – Suppl. Fig. 1 | HS + 10 μM capsaicin | 71 | -0.11669 | 0.09828 | 5 | 2 |
| 4 – Suppl. Fig. 1 | HS + 10 μM capsaicin | 72 | -0.1066 | 0.11007 | 5 | 2 |
| 4 – Suppl. Fig. 1 | HS + 10 μM capsaicin | 73 | -0.08365 | 0.11185 | 5 | 2 |
| 4 – Suppl. Fig. 1 | HS + 10 μM capsaicin | 74 | -0.06066 | 0.12293 | 5 | 2 |
| 4 – Suppl. Fig. 1 | HS + 10 μM capsaicin | 75 | -0.04121 | 0.11889 | 5 | 2 |
| 4 – Suppl. Fig. 1 | HS + 10 μM capsaicin | 76 | -0.05587 | 0.10333 | 5 | 2 |
| 4 – Suppl. Fig. 1 | HS + 10 μM capsaicin | 77 | -0.10125 | 0.10587 | 5 | 2 |
| 4 – Suppl. Fig. 1 | HS + 10 μM capsaicin | 78 | -0.11442 | 0.11006 | 5 | 2 |
| 4 – Suppl. Fig. 1 | HS + 10 μM capsaicin | 79 | -0.06941 | 0.11641 | 5 | 2 |
| 4 – Suppl. Fig. 1 | HS + 10 μM capsaicin | 80 | -0.06262 | 0.11525 | 5 | 2 |
| 4 – Suppl. Fig. 1 | HS + 10 μM capsaicin | 81 | -0.07046 | 0.11626 | 5 | 2 |
| 4 – Suppl. Fig. 1 | HS + 10 μM capsaicin | 82 | -0.08853 | 0.115 | 5 | 2 |
| 4 – Suppl. Fig. 1 | HS + 10 μM capsaicin | 83 | -0.08818 | 0.11604 | 5 | 2 |
| 4 – Suppl. Fig. 1 | HS + 10 μM capsaicin | 84 | -0.06502 | 0.11702 | 5 | 2 |
| 4 – Suppl. Fig. 1 | HS + 10 μM capsaicin | 85 | -0.10117 | 0.11462 | 5 | 2 |
| 4 – Suppl. Fig. 1 | HS + 10 μM capsaicin | 86 | -0.09632 | 0.12355 | 5 | 2 |
| 4 – Suppl. Fig. 1 | HS + 10 μM capsaicin | 87 | -0.12099 | 0.11307 | 5 | 2 |
| 4 – Suppl. Fig. 1 | HS + 10 μM capsaicin | 88 | -0.06395 | 0.1127 | 5 | 2 |
| 4 – Suppl. Fig. 1 | HS + 10 μM capsaicin | 89 | -0.05558 | 0.12243 | 5 | 2 |
| 4 – Suppl. Fig. 1 | HS + 10 μM capsaicin | 90 | -0.0892 | 0.1223 | 5 | 2 |
| 4 – Suppl. Fig. 1 | HS + 10 μM capsaicin | 91 | -0.06722 | 0.12786 | 5 | 2 |
| 4 – Suppl. Fig. 1 | HS + 10 μM capsaicin | 92 | -0.06155 | 0.1242 | 5 | 2 |
| 4 – Suppl. Fig. 1 | HS + 10 μM capsaicin | 93 | -0.05198 | 0.12786 | 5 | 2 |
| 4 – Suppl. Fig. 1 | HS + 10 μM capsaicin | 94 | -0.07337 | 0.13195 | 5 | 2 |
| 4 – Suppl. Fig. 1 | HS + 10 μM capsaicin | 95 | -0.04342 | 0.14251 | 5 | 2 |
| 4 – Suppl. Fig. 1 | HS + 10 μM capsaicin | 96 | -0.04133 | 0.14027 | 5 | 2 |
| 4 – Suppl. Fig. 1 | HS + 10 μM capsaicin | 97 | -0.07094 | 0.12817 | 5 | 2 |
| 4 – Suppl. Fig. 1 | HS + 10 μM capsaicin | 98 | -0.0209 | 0.13406 | 5 | 2 |
| 4 – Suppl. Fig. 1 | HS + 10 μM capsaicin | 99 | -0.05779 | 0.13792 | 5 | 2 |
| 4 – Suppl. Fig. 1 | HS + 10 μM capsaicin | 100 | -0.06662 | 0.13175 | 5 | 2 |
| 4 – Suppl. Fig. 1 | HS + 10 μM capsaicin | 101 | -0.05226 | 0.12951 | 5 | 2 |
| 4 – Suppl. Fig. 1 | HS + 10 μM capsaicin | 102 | -0.07415 | 0.13017 | 5 | 2 |
| 4 – Suppl. Fig. 1 | HS + 10 μM capsaicin | 103 | -0.07821 | 0.13234 | 5 | 2 |
| 4 – Suppl. Fig. 1 | HS + 10 μM capsaicin | 104 | -0.07447 | 0.13373 | 5 | 2 |
| 4 – Suppl. Fig. 1 | HS + 10 μM capsaicin | 105 | -0.08219 | 0.12897 | 5 | 2 |
| 4 – Suppl. Fig. 1 | HS + 10 μM capsaicin | 106 | -0.09162 | 0.12284 | 5 | 2 |
| 4 – Suppl. Fig. 1 | HS + 10 μM capsaicin | 107 | -0.07758 | 0.14242 | 5 | 2 |
| 4 – Suppl. Fig. 1 | HS + 10 μM capsaicin | 108 | -0.05167 | 0.12448 | 5 | 2 |
| 4 – Suppl. Fig. 1 | HS + 10 μM capsaicin | 109 | -0.10912 | 0.1283 | 5 | 2 |
| 4 – Suppl. Fig. 1 | HS + 10 μM capsaicin | 110 | -0.08135 | 0.13364 | 5 | 2 |
| 4 – Suppl. Fig. 1 | HS + 10 μM capsaicin | 111 | -0.08535 | 0.12533 | 5 | 2 |
| 4 – Suppl. Fig. 1 | HS + 10 μM capsaicin | 112 | -0.07089 | 0.13816 | 5 | 2 |
| 4 – Suppl. Fig. 1 | HS + 10 μM capsaicin | 113 | -0.07346 | 0.13188 | 5 | 2 |
| 4 – Suppl. Fig. 1 | HS + 10 μM capsaicin | 114 | -0.10181 | 0.13663 | 5 | 2 |
| 4 – Suppl. Fig. 1 | HS + 10 μM capsaicin | 115 | -0.09163 | 0.1375 | 5 | 2 |
| 4 – Suppl. Fig. 1 | HS + 10 μM capsaicin | 116 | -0.06812 | 0.12425 | 5 | 2 |
| 4 – Suppl. Fig. 1 | HS + 10 μM capsaicin | 117 | -0.08229 | 0.12951 | 5 | 2 |
| 4 – Suppl. Fig. 1 | HS + 10 μM capsaicin | 118 | -0.11175 | 0.12104 | 5 | 2 |
| 4 – Suppl. Fig. 1 | HS + 10 μM capsaicin | 119 | -0.0741 | 0.12761 | 5 | 2 |
| 4 – Suppl. Fig. 1 | HS + 10 μM capsaicin | 120 | -0.10501 | 0.14552 | 5 | 2 |
| 4 – Suppl. Fig. 1 | HS + 10 μM capsaicin | 121 | -0.114 | 0.13258 | 5 | 2 |
| 4 – Suppl. Fig. 1 | HS + 10 μM capsaicin | 122 | 0.03975 | 0.06103 | 5 | 2 |
| 4 – Suppl. Fig. 1 | HS + 10 μM capsaicin | 123 | 0.00721 | 0.05456 | 5 | 2 |
| 4 – Suppl. Fig. 1 | HS + 10 μM capsaicin | 124 | 0.06019 | 0.03978 | 5 | 2 |
| 4 – Suppl. Fig. 1 | HS + 10 μM capsaicin | 125 | 0.10654 | 0.03607 | 5 | 2 |
| 4 – Suppl. Fig. 1 | HS + 10 μM capsaicin | 126 | 0.07368 | 0.03479 | 5 | 2 |
| 4 – Suppl. Fig. 1 | HS + 10 μM capsaicin | 127 | 0.04196 | 0.01911 | 5 | 2 |
| 4 – Suppl. Fig. 1 | HS + 10 μM capsaicin | 128 | 0.02 | 0.02271 | 5 | 2 |
| 4 – Suppl. Fig. 1 | HS + 10 μM capsaicin | 129 | 0.0106 | 0.01356 | 5 | 2 |
| 4 – Suppl. Fig. 1 | HS + 10 μM capsaicin | 130 | 0.01194 | 0.01808 | 5 | 2 |
| 4 – Suppl. Fig. 1 | HS + 10 μM capsaicin | 131 | -0.03452 | 0.00932 | 5 | 2 |
| 4 – Suppl. Fig. 1 | HS + 10 μM capsaicin | 132 | 0.03559 | 0.01661 | 5 | 2 |
| 4 – Suppl. Fig. 1 | HS + 10 μM capsaicin | 133 | -4.10685E-4 | 0.02622 | 5 | 2 |
| 4 – Suppl. Fig. 1 | HS + 10 μM capsaicin | 134 | 0.00149 | 0.01572 | 5 | 2 |
| 4 – Suppl. Fig. 1 | HS + 10 μM capsaicin | 135 | -0.03309 | 0.03298 | 5 | 2 |
| 4 – Suppl. Fig. 1 | HS + 10 μM capsaicin | 136 | -0.05512 | 0.01627 | 5 | 2 |
| 4 – Suppl. Fig. 1 | HS + 10 μM capsaicin | 137 | -0.0177 | 0.02191 | 5 | 2 |
| 4 – Suppl. Fig. 1 | HS + 10 μM capsaicin | 138 | -0.05919 | 0.02893 | 5 | 2 |
| 4 – Suppl. Fig. 1 | HS + 10 μM capsaicin | 139 | -0.06283 | 0.04059 | 5 | 2 |
| 4 – Suppl. Fig. 1 | HS + 10 μM capsaicin | 140 | -0.06378 | 0.04294 | 5 | 2 |
| 4 – Suppl. Fig. 1 | HS + 10 μM capsaicin | 141 | -0.08231 | 0.02956 | 5 | 2 |
| 4 – Suppl. Fig. 1 | HS + 10 μM capsaicin | 142 | -0.04777 | 0.03526 | 5 | 2 |
| 4 – Suppl. Fig. 1 | HS + 10 μM capsaicin | 143 | -0.01727 | 0.04069 | 5 | 2 |
| 4 – Suppl. Fig. 1 | HS + 10 μM capsaicin | 144 | -0.06404 | 0.03937 | 5 | 2 |
| 4 – Suppl. Fig. 1 | HS + 10 μM capsaicin | 145 | -0.06452 | 0.03658 | 5 | 2 |
| 4 – Suppl. Fig. 1 | HS + 10 μM capsaicin | 146 | -0.0975 | 0.04942 | 5 | 2 |
| 4 – Suppl. Fig. 1 | HS + 10 μM capsaicin | 147 | -0.07044 | 0.05779 | 5 | 2 |
| 4 – Suppl. Fig. 1 | HS + 10 μM capsaicin | 148 | -0.04543 | 0.05984 | 5 | 2 |
| 4 – Suppl. Fig. 1 | HS + 10 μM capsaicin | 149 | -0.05921 | 0.06062 | 5 | 2 |
| 4 – Suppl. Fig. 1 | HS + 10 μM capsaicin | 150 | -0.0911 | 0.05967 | 5 | 2 |

**(D) Calcium imaging (stimulation with 500 μM carvacrol after 20 s)**

| Fig. no | Bath solution | Time (s) | Mean ΔF/F_0_ | SEM of mean ΔF/F_0_ | n, no. of cells | No. of donors |
| --- | --- | --- | --- | --- | --- | --- |
| 4 – Suppl. Fig. 1 | HS | 1 | 0.02611 | 0.0087 | 4 | 2 |
| 4 – Suppl. Fig. 1 | HS | 2 | 0.01223 | 0.00896 | 4 | 2 |
| 4 – Suppl. Fig. 1 | HS | 3 | 0.01347 | 0.01809 | 4 | 2 |
| 4 – Suppl. Fig. 1 | HS | 4 | 0.00355 | 0.01958 | 4 | 2 |
| 4 – Suppl. Fig. 1 | HS | 5 | -0.00728 | 0.01405 | 4 | 2 |
| 4 – Suppl. Fig. 1 | HS | 6 | 0.00107 | 0.00864 | 4 | 2 |
| 4 – Suppl. Fig. 1 | HS | 7 | -0.03023 | 0.01495 | 4 | 2 |
| 4 – Suppl. Fig. 1 | HS | 8 | -0.01932 | 0.00625 | 4 | 2 |
| 4 – Suppl. Fig. 1 | HS | 9 | 0.01245 | 0.00747 | 4 | 2 |
| 4 – Suppl. Fig. 1 | HS | 10 | 0.00215 | 0.02058 | 4 | 2 |
| 4 – Suppl. Fig. 1 | HS | 11 | -0.00592 | 0.00878 | 4 | 2 |
| 4 – Suppl. Fig. 1 | HS | 12 | 0.01013 | 0.00422 | 4 | 2 |
| 4 – Suppl. Fig. 1 | HS | 13 | -0.00481 | 0.00918 | 4 | 2 |
| 4 – Suppl. Fig. 1 | HS | 14 | 0.00993 | 0.01069 | 4 | 2 |
| 4 – Suppl. Fig. 1 | HS | 15 | -0.00147 | 0.00623 | 4 | 2 |
| 4 – Suppl. Fig. 1 | HS | 16 | -0.01141 | 0.0153 | 4 | 2 |
| 4 – Suppl. Fig. 1 | HS | 17 | -0.01621 | 0.01191 | 4 | 2 |
| 4 – Suppl. Fig. 1 | HS | 18 | 0.01357 | 0.01435 | 4 | 2 |
| 4 – Suppl. Fig. 1 | HS | 19 | -0.00782 | 0.01536 | 4 | 2 |
| 4 – Suppl. Fig. 1 | HS | 20 | -1.96844E-4 | 0.02032 | 4 | 2 |
| 4 – Suppl. Fig. 1 | HS + 500 μM carvacrol | 21 | 7.59213E-4 | 0.02804 | 4 | 2 |
| 4 – Suppl. Fig. 1 | HS + 500 μM carvacrol | 22 | -0.02029 | 0.04359 | 4 | 2 |
| 4 – Suppl. Fig. 1 | HS + 500 μM carvacrol | 23 | -0.01146 | 0.02521 | 4 | 2 |
| 4 – Suppl. Fig. 1 | HS + 500 μM carvacrol | 24 | 0.00529 | 0.03772 | 4 | 2 |
| 4 – Suppl. Fig. 1 | HS + 500 μM carvacrol | 25 | -0.01162 | 0.02524 | 4 | 2 |
| 4 – Suppl. Fig. 1 | HS + 500 μM carvacrol | 26 | -0.00968 | 0.02087 | 4 | 2 |
| 4 – Suppl. Fig. 1 | HS + 500 μM carvacrol | 27 | -0.02563 | 0.02303 | 4 | 2 |
| 4 – Suppl. Fig. 1 | HS + 500 μM carvacrol | 28 | -0.01989 | 0.01571 | 4 | 2 |
| 4 – Suppl. Fig. 1 | HS + 500 μM carvacrol | 29 | -0.00707 | 0.0258 | 4 | 2 |
| 4 – Suppl. Fig. 1 | HS + 500 μM carvacrol | 30 | -0.01979 | 0.04289 | 4 | 2 |
| 4 – Suppl. Fig. 1 | HS + 500 μM carvacrol | 31 | -0.02946 | 0.04457 | 4 | 2 |
| 4 – Suppl. Fig. 1 | HS + 500 μM carvacrol | 32 | -0.01488 | 0.03003 | 4 | 2 |
| 4 – Suppl. Fig. 1 | HS + 500 μM carvacrol | 33 | -0.01083 | 0.04081 | 4 | 2 |
| 4 – Suppl. Fig. 1 | HS + 500 μM carvacrol | 34 | -0.00797 | 0.03638 | 4 | 2 |
| 4 – Suppl. Fig. 1 | HS + 500 μM carvacrol | 35 | -0.05372 | 0.03209 | 4 | 2 |
| 4 – Suppl. Fig. 1 | HS + 500 μM carvacrol | 36 | -0.03504 | 0.02784 | 4 | 2 |
| 4 – Suppl. Fig. 1 | HS + 500 μM carvacrol | 37 | -0.04594 | 0.04206 | 4 | 2 |
| 4 – Suppl. Fig. 1 | HS + 500 μM carvacrol | 38 | -0.01355 | 0.03186 | 4 | 2 |
| 4 – Suppl. Fig. 1 | HS + 500 μM carvacrol | 39 | -0.02945 | 0.03385 | 4 | 2 |
| 4 – Suppl. Fig. 1 | HS + 500 μM carvacrol | 40 | -0.03044 | 0.0335 | 4 | 2 |
| 4 – Suppl. Fig. 1 | HS + 500 μM carvacrol | 41 | -0.04633 | 0.04159 | 4 | 2 |
| 4 – Suppl. Fig. 1 | HS + 500 μM carvacrol | 42 | -0.06416 | 0.03565 | 4 | 2 |
| 4 – Suppl. Fig. 1 | HS + 500 μM carvacrol | 43 | -0.04557 | 0.05487 | 4 | 2 |
| 4 – Suppl. Fig. 1 | HS + 500 μM carvacrol | 44 | -0.07809 | 0.04713 | 4 | 2 |
| 4 – Suppl. Fig. 1 | HS + 500 μM carvacrol | 45 | -0.06335 | 0.04351 | 4 | 2 |
| 4 – Suppl. Fig. 1 | HS + 500 μM carvacrol | 46 | -0.07315 | 0.05848 | 4 | 2 |
| 4 – Suppl. Fig. 1 | HS + 500 μM carvacrol | 47 | -0.06251 | 0.04965 | 4 | 2 |
| 4 – Suppl. Fig. 1 | HS + 500 μM carvacrol | 48 | -0.063 | 0.06561 | 4 | 2 |
| 4 – Suppl. Fig. 1 | HS + 500 μM carvacrol | 49 | -0.06063 | 0.0577 | 4 | 2 |
| 4 – Suppl. Fig. 1 | HS + 500 μM carvacrol | 50 | -0.06031 | 0.05406 | 4 | 2 |
| 4 – Suppl. Fig. 1 | HS + 500 μM carvacrol | 51 | -0.07601 | 0.05504 | 4 | 2 |
| 4 – Suppl. Fig. 1 | HS + 500 μM carvacrol | 52 | -0.05048 | 0.05046 | 4 | 2 |
| 4 – Suppl. Fig. 1 | HS + 500 μM carvacrol | 53 | -0.06674 | 0.0657 | 4 | 2 |
| 4 – Suppl. Fig. 1 | HS + 500 μM carvacrol | 54 | -0.08216 | 0.06814 | 4 | 2 |
| 4 – Suppl. Fig. 1 | HS + 500 μM carvacrol | 55 | -0.08507 | 0.05274 | 4 | 2 |
| 4 – Suppl. Fig. 1 | HS + 500 μM carvacrol | 56 | -0.09597 | 0.05718 | 4 | 2 |
| 4 – Suppl. Fig. 1 | HS + 500 μM carvacrol | 57 | -0.07759 | 0.05734 | 4 | 2 |
| 4 – Suppl. Fig. 1 | HS + 500 μM carvacrol | 58 | -0.06625 | 0.06593 | 4 | 2 |
| 4 – Suppl. Fig. 1 | HS + 500 μM carvacrol | 59 | -0.05452 | 0.06535 | 4 | 2 |
| 4 – Suppl. Fig. 1 | HS + 500 μM carvacrol | 60 | -0.06403 | 0.05647 | 4 | 2 |
| 4 – Suppl. Fig. 1 | HS + 500 μM carvacrol | 61 | -0.05069 | 0.07044 | 4 | 2 |
| 4 – Suppl. Fig. 1 | HS + 500 μM carvacrol | 62 | -0.06107 | 0.06911 | 4 | 2 |
| 4 – Suppl. Fig. 1 | HS + 500 μM carvacrol | 63 | -0.0597 | 0.06393 | 4 | 2 |
| 4 – Suppl. Fig. 1 | HS + 500 μM carvacrol | 64 | -0.05884 | 0.07059 | 4 | 2 |
| 4 – Suppl. Fig. 1 | HS + 500 μM carvacrol | 65 | -0.09007 | 0.06706 | 4 | 2 |
| 4 – Suppl. Fig. 1 | HS + 500 μM carvacrol | 66 | -0.05405 | 0.08224 | 4 | 2 |
| 4 – Suppl. Fig. 1 | HS + 500 μM carvacrol | 67 | -0.03703 | 0.07157 | 4 | 2 |
| 4 – Suppl. Fig. 1 | HS + 500 μM carvacrol | 68 | -0.07639 | 0.07483 | 4 | 2 |
| 4 – Suppl. Fig. 1 | HS + 500 μM carvacrol | 69 | -0.05218 | 0.06695 | 4 | 2 |
| 4 – Suppl. Fig. 1 | HS + 500 μM carvacrol | 70 | -0.07747 | 0.05506 | 4 | 2 |
| 4 – Suppl. Fig. 1 | HS + 500 μM carvacrol | 71 | -0.10773 | 0.07429 | 4 | 2 |
| 4 – Suppl. Fig. 1 | HS + 500 μM carvacrol | 72 | -0.07225 | 0.0603 | 4 | 2 |
| 4 – Suppl. Fig. 1 | HS + 500 μM carvacrol | 73 | -0.07004 | 0.06216 | 4 | 2 |
| 4 – Suppl. Fig. 1 | HS + 500 μM carvacrol | 74 | -0.0537 | 0.07356 | 4 | 2 |
| 4 – Suppl. Fig. 1 | HS + 500 μM carvacrol | 75 | -0.06564 | 0.07432 | 4 | 2 |
| 4 – Suppl. Fig. 1 | HS + 500 μM carvacrol | 76 | -0.07898 | 0.07454 | 4 | 2 |
| 4 – Suppl. Fig. 1 | HS + 500 μM carvacrol | 77 | -0.06851 | 0.07589 | 4 | 2 |
| 4 – Suppl. Fig. 1 | HS + 500 μM carvacrol | 78 | -0.07537 | 0.07262 | 4 | 2 |
| 4 – Suppl. Fig. 1 | HS + 500 μM carvacrol | 79 | -0.05275 | 0.07888 | 4 | 2 |
| 4 – Suppl. Fig. 1 | HS + 500 μM carvacrol | 80 | -0.0787 | 0.08625 | 4 | 2 |
| 4 – Suppl. Fig. 1 | HS + 500 μM carvacrol | 81 | -0.06351 | 0.06505 | 4 | 2 |
| 4 – Suppl. Fig. 1 | HS + 500 μM carvacrol | 82 | -0.06741 | 0.08443 | 4 | 2 |
| 4 – Suppl. Fig. 1 | HS + 500 μM carvacrol | 83 | -0.05303 | 0.09118 | 4 | 2 |
| 4 – Suppl. Fig. 1 | HS + 500 μM carvacrol | 84 | -0.0513 | 0.08246 | 4 | 2 |
| 4 – Suppl. Fig. 1 | HS + 500 μM carvacrol | 85 | -0.07337 | 0.08359 | 4 | 2 |
| 4 – Suppl. Fig. 1 | HS + 500 μM carvacrol | 86 | -0.05513 | 0.07934 | 4 | 2 |
| 4 – Suppl. Fig. 1 | HS + 500 μM carvacrol | 87 | -0.05696 | 0.09363 | 4 | 2 |
| 4 – Suppl. Fig. 1 | HS + 500 μM carvacrol | 88 | -0.06417 | 0.08183 | 4 | 2 |
| 4 – Suppl. Fig. 1 | HS + 500 μM carvacrol | 89 | -0.06226 | 0.08991 | 4 | 2 |
| 4 – Suppl. Fig. 1 | HS + 500 μM carvacrol | 90 | -0.0589 | 0.08076 | 4 | 2 |
| 4 – Suppl. Fig. 1 | HS + 500 μM carvacrol | 91 | -0.0913 | 0.09009 | 4 | 2 |
| 4 – Suppl. Fig. 1 | HS + 500 μM carvacrol | 92 | -0.06323 | 0.08253 | 4 | 2 |
| 4 – Suppl. Fig. 1 | HS + 500 μM carvacrol | 93 | -0.05592 | 0.08494 | 4 | 2 |
| 4 – Suppl. Fig. 1 | HS + 500 μM carvacrol | 94 | -0.071 | 0.09826 | 4 | 2 |
| 4 – Suppl. Fig. 1 | HS + 500 μM carvacrol | 95 | -0.04909 | 0.08736 | 4 | 2 |
| 4 – Suppl. Fig. 1 | HS + 500 μM carvacrol | 96 | -0.07477 | 0.09408 | 4 | 2 |
| 4 – Suppl. Fig. 1 | HS + 500 μM carvacrol | 97 | -0.05238 | 0.08816 | 4 | 2 |
| 4 – Suppl. Fig. 1 | HS + 500 μM carvacrol | 98 | -0.05204 | 0.08104 | 4 | 2 |
| 4 – Suppl. Fig. 1 | HS + 500 μM carvacrol | 99 | -0.05634 | 0.08761 | 4 | 2 |
| 4 – Suppl. Fig. 1 | HS + 500 μM carvacrol | 100 | -0.05133 | 0.09144 | 4 | 2 |
| 4 – Suppl. Fig. 1 | HS + 500 μM carvacrol | 101 | -0.06662 | 0.08943 | 4 | 2 |
| 4 – Suppl. Fig. 1 | HS + 500 μM carvacrol | 102 | -0.05556 | 0.09322 | 4 | 2 |
| 4 – Suppl. Fig. 1 | HS + 500 μM carvacrol | 103 | -0.08913 | 0.09604 | 4 | 2 |
| 4 – Suppl. Fig. 1 | HS + 500 μM carvacrol | 104 | -0.05849 | 0.10142 | 4 | 2 |
| 4 – Suppl. Fig. 1 | HS + 500 μM carvacrol | 105 | -0.04656 | 0.09019 | 4 | 2 |
| 4 – Suppl. Fig. 1 | HS + 500 μM carvacrol | 106 | -0.06797 | 0.09195 | 4 | 2 |
| 4 – Suppl. Fig. 1 | HS + 500 μM carvacrol | 107 | -0.07906 | 0.08876 | 4 | 2 |
| 4 – Suppl. Fig. 1 | HS + 500 μM carvacrol | 108 | -0.04687 | 0.09225 | 4 | 2 |
| 4 – Suppl. Fig. 1 | HS + 500 μM carvacrol | 109 | -0.04592 | 0.09444 | 4 | 2 |
| 4 – Suppl. Fig. 1 | HS + 500 μM carvacrol | 110 | -0.0507 | 0.09978 | 4 | 2 |
| 4 – Suppl. Fig. 1 | HS + 500 μM carvacrol | 111 | -0.04389 | 0.10266 | 4 | 2 |
| 4 – Suppl. Fig. 1 | HS + 500 μM carvacrol | 112 | -0.02342 | 0.10111 | 4 | 2 |
| 4 – Suppl. Fig. 1 | HS + 500 μM carvacrol | 113 | -0.00591 | 0.11139 | 4 | 2 |
| 4 – Suppl. Fig. 1 | HS + 500 μM carvacrol | 114 | 0.00132 | 0.11443 | 4 | 2 |
| 4 – Suppl. Fig. 1 | HS + 500 μM carvacrol | 115 | 0.00498 | 0.12077 | 4 | 2 |
| 4 – Suppl. Fig. 1 | HS + 500 μM carvacrol | 116 | 0.00605 | 0.11994 | 4 | 2 |
| 4 – Suppl. Fig. 1 | HS + 500 μM carvacrol | 117 | 0.0232 | 0.12555 | 4 | 2 |
| 4 – Suppl. Fig. 1 | HS + 500 μM carvacrol | 118 | 0.00102 | 0.11497 | 4 | 2 |
| 4 – Suppl. Fig. 1 | HS + 500 μM carvacrol | 119 | 0.03712 | 0.12008 | 4 | 2 |
| 4 – Suppl. Fig. 1 | HS + 500 μM carvacrol | 120 | -0.03191 | 0.11782 | 4 | 2 |
| 4 – Suppl. Fig. 1 | HS + 500 μM carvacrol | 121 | -0.0092 | 0.10169 | 4 | 2 |
| 4 – Suppl. Fig. 1 | HS + 500 μM carvacrol | 122 | -0.02125 | 0.10592 | 4 | 2 |
| 4 – Suppl. Fig. 1 | HS + 500 μM carvacrol | 123 | -0.01808 | 0.09896 | 4 | 2 |
| 4 – Suppl. Fig. 1 | HS + 500 μM carvacrol | 124 | -0.01204 | 0.10236 | 4 | 2 |
| 4 – Suppl. Fig. 1 | HS + 500 μM carvacrol | 125 | -0.01202 | 0.0999 | 4 | 2 |
| 4 – Suppl. Fig. 1 | HS + 500 μM carvacrol | 126 | -0.01095 | 0.09147 | 4 | 2 |
| 4 – Suppl. Fig. 1 | HS + 500 μM carvacrol | 127 | -0.03109 | 0.09687 | 4 | 2 |
| 4 – Suppl. Fig. 1 | HS + 500 μM carvacrol | 128 | 0.00283 | 0.1006 | 4 | 2 |
| 4 – Suppl. Fig. 1 | HS + 500 μM carvacrol | 129 | -0.02938 | 0.09745 | 4 | 2 |
| 4 – Suppl. Fig. 1 | HS + 500 μM carvacrol | 130 | -0.04798 | 0.09407 | 4 | 2 |
| 4 – Suppl. Fig. 1 | HS + 500 μM carvacrol | 131 | -0.04375 | 0.09783 | 4 | 2 |
| 4 – Suppl. Fig. 1 | HS + 500 μM carvacrol | 132 | -0.03021 | 0.09263 | 4 | 2 |
| 4 – Suppl. Fig. 1 | HS + 500 μM carvacrol | 133 | -0.01546 | 0.10389 | 4 | 2 |
| 4 – Suppl. Fig. 1 | HS + 500 μM carvacrol | 134 | -0.00751 | 0.09845 | 4 | 2 |
| 4 – Suppl. Fig. 1 | HS + 500 μM carvacrol | 135 | -0.04126 | 0.09977 | 4 | 2 |
| 4 – Suppl. Fig. 1 | HS + 500 μM carvacrol | 136 | -0.029 | 0.09441 | 4 | 2 |
| 4 – Suppl. Fig. 1 | HS + 500 μM carvacrol | 137 | -0.04065 | 0.09682 | 4 | 2 |
| 4 – Suppl. Fig. 1 | HS + 500 μM carvacrol | 138 | -0.03879 | 0.10161 | 4 | 2 |
| 4 – Suppl. Fig. 1 | HS + 500 μM carvacrol | 139 | -0.02022 | 0.09036 | 4 | 2 |
| 4 – Suppl. Fig. 1 | HS + 500 μM carvacrol | 140 | -0.03357 | 0.10031 | 4 | 2 |
| 4 – Suppl. Fig. 1 | HS + 500 μM carvacrol | 141 | -0.04109 | 0.10352 | 4 | 2 |
| 4 – Suppl. Fig. 1 | HS + 500 μM carvacrol | 142 | -0.04802 | 0.10613 | 4 | 2 |
| 4 – Suppl. Fig. 1 | HS + 500 μM carvacrol | 143 | -0.05031 | 0.09871 | 4 | 2 |
| 4 – Suppl. Fig. 1 | HS + 500 μM carvacrol | 144 | -0.0407 | 0.10194 | 4 | 2 |
| 4 – Suppl. Fig. 1 | HS + 500 μM carvacrol | 145 | -0.02139 | 0.0983 | 4 | 2 |
| 4 – Suppl. Fig. 1 | HS + 500 μM carvacrol | 146 | -0.03326 | 0.09508 | 4 | 2 |
| 4 – Suppl. Fig. 1 | HS + 500 μM carvacrol | 147 | -0.02913 | 0.09884 | 4 | 2 |
| 4 – Suppl. Fig. 1 | HS + 500 μM carvacrol | 148 | -0.00234 | 0.10422 | 4 | 2 |
| 4 – Suppl. Fig. 1 | HS + 500 μM carvacrol | 149 | -0.02471 | 0.10371 | 4 | 2 |
| 4 – Suppl. Fig. 1 | HS + 500 μM carvacrol | 150 | 0.00224 | 0.10177 | 4 | 2 |

**(E) Pregnonolone sulfate effect on I_DSper_**

| Fig. no | Experimental condition | I at -80 mV normalized to control | I at +80 mV normalized to control | n, no. of cells | No. of donors |
| --- | --- | --- | --- | --- | --- |
| 4 – Suppl. Fig. 1 | DSper control | -1 | 1 | 7 | 3 |
| 4 – Suppl. Fig. 1 | DSper + 10 μM PS | -1.04058 ± 0.05 | 1.11964 ± 0.12 | 7 | 3 |
