# Supplementary material for: TRPV4 is the temperature-sensitive ion channel of human sperm
Source: eLife. 2018 Jul 2;7:e35853. doi: 10.7554/eLife.35853 (PMC6051745; doi:10.7554/eLife.35853)
Supplement: Figure 5—source data 1. — (A) DSper’s temperature-sensitivity in presence of TRPV4 specific inhibitors RN1734 and HC067047. (B) EtOH vehicle control. [file elife-35853-fig5-data1.docx]

Source File: Figure 5

**(A) DSper’s temperature-sensitivity in presence of TRPV4 inhibitors RN1734 and HC067047**

| Fig. no | Experimental condition | I at +80 mV, normalized to 22 ̊C | n, no. of cells | No. of donors |
| --- | --- | --- | --- | --- |
| 5 A-B | DSper control, 22 ̊C | 1 | 11 | 3 |
| 5 A-B | DSper control, 24 ̊C | 1.44179 ± 0.08 | 11 | 3 |
| 5 A-B | DSper control, 26 ̊C | 1.7496 ± 0.12 | 10 | 3 |
| 5 A-B | DSper control,  28 ̊C | 2.04909 ± 0.14 | 11 | 3 |
| 5 A-B | DSper control, 30 ̊C | 2.34425 ± 0.21 | 10 | 3 |
| 5 A-B | DSper control, 32 ̊C | 2.42261 ± 0.31 | 7 | 3 |
| 5 A-B | DSper control, 34 ̊C | 2.92612 ± 0.37 | 5 | 3 |
| 5 A-B | DSper control, 36 ̊C | 2.58409 ± 0.30 | 5 | 3 |
| 5 A-B | DSper control, 38 ̊C | 3.09036 ± 0.42 | 3 | 3 |
| 5 A-B | DSper control, 40 ̊C | 3.18489 ± 0.54 | 4 | 3 |
| 5 A | DSper + 5 μM RN1734, 22 ̊C | 1 | 9 | 3 |
| 5 A | DSper + 5 μM RN1734, 24 ̊C | 0.97062 ± 0.09 | 9 | 3 |
| 5 A | DSper + 5 μM RN1734, 26 ̊C | 1.05494 ± 0.16 | 8 | 3 |
| 5 A | DSper + 5 μM RN1734, 28 ̊C | 1.15186 ± 0.27 | 8 | 3 |
| 5 A | DSper + 5 μM RN1734, 30 ̊C | 1.19493 ± 0.35 | 8 | 3 |
| 5 A | DSper + 5 μM RN1734, 32 ̊C | 1.18669 ± 0.35 | 4 | 3 |
| 5 A | DSper + 5 μM RN1734, 34 ̊C | 1.06506 ± 0.21 | 4 | 3 |
| 5 A | DSper + 5 μM RN1734, 36 ̊C | 1.17266 ± 0.30 | 3 | 3 |
| 5 A | DSper + 5 μM RN1734, 38 ̊C | 1.16951 ± 0.23 | 2 | 3 |
| 5 A | DSper + 5 μM RN1734, 40 ̊C | 1.17135 ± 0.25 | 3 | 3 |
| 5 B | DSper + 1 μM HC067047, 22 ̊C | 1 | 8 | 3 |
| 5 B | DSper + 1 μM HC067047, 24 ̊C | 0.95669 ± 0.03 | 7 | 3 |
| 5 B | DSper + 1 μM HC067047, 26 ̊C | 1.02706 ± 0.05 | 7 | 3 |
| 5 B | DSper + 1 μM HC067047, 28 ̊C | 1.07445 ± 0.06 | 7 | 3 |
| 5 B | DSper + 1 μM HC067047, 30 ̊C | 1.15395 ± 0.10 | 5 | 3 |
| 5 B | DSper + 1 μM HC067047, 32 ̊C | 1.12862 ± 0.10 | 5 | 3 |
| 5 B | DSper + 1 μM HC067047, 34 ̊C | 1.18305 ± 0.14 | 6 | 3 |
| 5 B | DSper + 1 μM HC067047, 36 ̊C | 1.1491 ± 0.26 | 4 | 3 |
| 5 B | DSper + 1 μM HC067047, 38 ̊C | 1.0377 ± 0.27 | 3 | 3 |
| 5 B | DSper + 1 μM HC067047, 40 ̊C | 1.00512 ± 0.14 | 3 | 3 |

**(B) EtOH vehicle control**

| Fig. no | Experimental condition | I at -80 mV normalized to 22 ˚C | I at +80 mV normalized to 22 ˚C | n, no. of cells | No. of donors |
| --- | --- | --- | --- | --- | --- |
| 5 – Suppl. Fig. 1 | DSper control, 22 ˚C | -1 | 1 | 5 | 2 |
| 5 – Suppl. Fig. 1 | DSper control, 34 ˚C | -2.92612 ± 0.37 | 1.65379 ± 0.10 | 5 | 2 |
| 5 – Suppl. Fig. 1 | DSper + 0.1 % EtOH, 22 ˚C | -1 | 1 | 3 | 2 |
| 5 – Suppl. Fig. 1 | DSper + 0.1 % EtOH, 34 ˚C | -2.82334 ± 0.16 | 1.9354 ± 0.32 | 3 | 2 |
