# Supplementary material for: TRPV4 is the temperature-sensitive ion channel of human sperm
Source: eLife. 2018 Jul 2;7:e35853. doi: 10.7554/eLife.35853 (PMC6051745; doi:10.7554/eLife.35853)
Supplement: Figure 6—source data 1. [file elife-35853-fig6-data1.docx]

Source File: Figure 6

**TRPV4 activity recombinantly expressed in HEK293 cells**

| Fig. no | Experimental condition | I at -80 mV, pA/pF | I at +80 mV, pA/pF | I at -80 mV normalized to control | I at +80 mV normalized to control | n, no. of cells |
| --- | --- | --- | --- | --- | --- | --- |
| 6 C | nontransfected cells (22 ̊C) | -0.7102 ± 0.20 | 3.4922 ± 0.74 |  |  | 11 |
| 6 D | nontransfected cells (24 ̊C) | -1.09558 ± 0.26 | 3.73541 ± 1.05 |  |  | 6 |
| 6 D | nontransfected cells (26 ̊C) | -0.9817 ± 0.29 | 4.62412 ± 1.52 |  |  | 6 |
| 6 D | nontransfected cells (28 ̊C) | -1.27588 ± 0.30 | 4.99232 ± 1.42 |  |  | 6 |
| 6 D | nontransfected cells (30 ̊C) | -1.6516 ± 0.34 | 5.33867 ± 1.87 |  |  | 4 |
| 6 D | nontransfected cells (32 ̊C) | -1.84993 ± 0.38 | 5.5726 ± 1.54 |  |  | 4 |
| 6 D | nontransfected cells (34 ̊C) | -2.03877 ± 0.70 | 4.7753 ± 0.14 |  |  | 3 |
| 6 D | nontransfected cells (36 ̊C) | -1.38143 ± 0.55 | 4.80864 ± 0.58 |  |  | 3 |
| 6 D | nontransfected cells (38 ̊C) | -1.6138 ± 0.19 | 5.11078 ± 1.09 |  |  | 3 |
| 6 D | nontransfected cells (40 ̊C) | -1.48792 ± 0.48 | 6.44084 ± 0.37 |  |  | 3 |
| 6 D | TRPV4 transfected cells (22 ̊C) | -31.87532 ± 10.63 | 55.79056 ± 13.83 |  |  | 8 |
| 6 D | TRPV4 transfected cells (24 ̊C) | -44.36327 ± 17.80 | 49.1603 ± 12.18 |  |  | 5 |
| 6 D | TRPV4 transfected cells (26 ̊C) | -45.39836 ± 14.24 | 57.88657 ± 13.50 |  |  | 5 |
| 6 D | TRPV4 transfected cells (28 ̊C) | -49.13446 ± 12.71 | 58.45959 ± 14.26 |  |  | 5 |
| 6 D | TRPV4 transfected cells (30 ̊C) | -64.35034 ± 13.90 | 86.07988 ± 6.85 |  |  | 4 |
| 6 D | TRPV4 transfected cells (32 ̊C) | -65.38725 ± 15.06 | 98.5871 ± 4.92 |  |  | 4 |
| 6 D | TRPV4 transfected cells (34 ̊C) | -81.14815 ± 16.01 | 97.04678 ± 8.65 |  |  | 4 |
| 6 D | TRPV4 transfected cells (36 ̊C) | -81.44113 ± 17.27 | 96.83105 ± 5.69 |  |  | 4 |
| 6 D | TRPV4 transfected cells (38 ̊C) | -82.271 ± 18.87 | 94.84634 ± 6.76 |  |  | 4 |
| 6 D | TRPV4 transfected cells (40 ̊C) | -90.14526 ± 22.73 | 93.15675 ± 11.79 |  |  | 4 |
| 6 G | nontransfected cells, control |  |  | -1 | 1 | 4 |
| 6 G | nontransfected cells + 10 μM RN1747 |  |  | -1.29506 ± 0.12 | 1.0832 ± 0.06 | 4 |
| 6 G | TRPV4 transfected cells, control |  |  | -1 | 1 | 4 |
| 6 G | TRPV4 transfected cells + 10 μM RN1747 |  |  | -50.67478 ± 10.32 | 35.83515 ± 4.85 | 4 |
